# Individual, household, and community-level determinants of undernutrition among pregnant women in the northern zone of the Sidama region, Ethiopia: A multi-level modified Poisson regression analysis

**DOI:** 10.1371/journal.pone.0315681

**Published:** 2024-12-17

**Authors:** Amanuel Yoseph, Lakew Mussie, Mehretu Belayneh

**Affiliations:** 1 School of Public Health, College of Medicine and Health Sciences, Hawassa University, Hawassa, Ethiopia; 2 Adare General Hospital, Hawassa City Administration, Adare, Hawassa; PLOS: Public Library of Science, ETHIOPIA

## Abstract

**Introduction:**

In Ethiopia, maternal undernutrition is a major public health concern. However, comprehensive evidence is lacking in the southern part of Ethiopia, specifically the household and community-level related determinants of undernutrition. Besides, the evidence about the prevalence and determinants of undernutrition is not yet documented in the current study setting. Thus, this study aimed to determine the prevalence of undernutrition and identify its determinants among pregnant women in Hawela Lida district of the Sidama region, Ethiopia.

**Methods:**

A community-based cross-sectional study was conducted on a sample of 515 pregnant women from June 1–25, 2024. A multi-stage sampling method was utilized to select eligible pregnant women. We collected data using a structured and pretested interviewer-administrated questionnaire and an anthropometric measurement. Data were collected using the Open Data Kit smart phone device and exported it to Stata version 17 for further processing and analysis. A multi-level mixed-effects modified Poisson regression analysis with robust variance was used to account for confounders and between and with cluster effects.

**Result:**

The prevalence of undernutrition among pregnant women was 41.7% (95% CI: 37.3–45.6). The prevalence of undernutrition was associated with planned pregnancy (adjusted prevalence ratio [APR]: 0.80; 95% CI: 0.66–0.98), household food insecurity (APR: 1.64; 95% CI: 1.26–2.13), inadequate dietary diversity (APR: 1.79; 95% CI: 1.43–2.25), and women’s poor knowledge of nutrition (APR: 1.68; 95% CI: 1.32–2.12) at individual levels. The identified determinants of undernutrition at the community level were low community literacy rates (APR: 4.62; 95% CI: 1.13–18.79) and low community wealth status (APR: 1.91; 95% CI: 1.10–3.31).

**Conclusion:**

Two in five pregnant women had an undernutrition problem in the study setting. Individual and community-level determinants contributed to the high prevalence of undernutrition. Thus, any prevention and control approaches to undernutrition should consider inter-sectorial collaboration to account for determinants at various levels. Besides, any program must emphasize the delivery of nutrition education about dietary diversity, particularly targeting pregnant mothers who have poor knowledge of nutrition and unplanned pregnancy at the individual level. Moreover, creating a small business reform for the community with low wealth status using agricultural extension workers must be considered.

## Introduction

Undernutrition is defined as a lack of the required calories and an overall insufficient intake of food and nutrients to meet a person’s needs and maintain good health [1]. Furthermore, undernutrition was caused by a combination of elevated nutritional demands and insufficient food consumption during pregnancy due to normal physiological change [2]. Undernutrition during pregnancy has significantly contributed to negative birth outcomes, maternal morbidity, and mortality [3].

Globally, the prevalence of undernutrition remains a serious public health concern, with 10% of pregnant women developing undernutrition [4]. The burden of maternal undernutrition is disproportionately high in Southeast Asia and Africa, as per reports from different studies due to poverty, political instability and civil war, food insecurity, inadequate dietary intake because of cultural taboos during pregnancy, and frequent infections [5–7]. For instance, a systematic review and meta-analysis reported that the pooled prevalence of undernutrition among pregnant women was 23.5% in Africa [5]. Besides, small-pocket studies reported a very high and variable prevalence of undernutrition among pregnant women in Africa, such as in Kenya (39.7%) [8], Ghana (28.8%) [9], and Ethiopia (47.9%) [10]. The Ethiopian Demographic and Health Survey (EDHS) report showed that maternal undernutrition decreased from 30% to 22% between 2000 and 2016. However, the rate of decline was slow, and urban and rural disparities in the prevalence of undernutrition persisted for decades [11]. Similarly, our comprehensive review of the literature showed a huge disparity in the prevalence of undernutrition among pregnant women in different regions of Ethiopia, with the smallest in the Gonder hospital (14.4%) [12] and the highest in the Haramaya district of eastern Ethiopia (47.9%) [10].

High prevalence of undernutrition in pregnant women’s has serious consequences such as morbidity, mortality, poor pregnancy and birth outcomes such as intrauterine fetal growth retardation (IUGR) and death, stillbirth, low birth weight, premature and neonatal mortality [13–15]. Also, research findings suggest that being vulnerable to undernutrition during pregnancy is associated with stunted growth and development in childhood, reduced intellectual and learning capacity, small stature in adulthood, lower academic attainment, and lower economic production [16, 17]. Moreover, it has intergenerational effects, and malnutrition can be passed down from mother to child. If they are girls, they are more likely to become undernourished mothers, and the vicious cycle repeats [18, 19].

Several determinants contribute to the high prevalence of undernutrition among pregnant women and can be categorized as distal (socio-demographic, economic, ecological, political, private, and public sector action), underlying (household food security with palatable and safe drinking water, appropriate dietary practice, food hygiene and safety, and nutrition education), and immediate (adequate dietary intake, good care, and infection) [20]. Studies also showed place of residence, literacy level, maternal age and age at first marriage, maternal health service use, meal skipping, meal frequency, maternal nutritional knowledge and attitude, mass media use, and socio-cultural food taboos contributed to the high prevalence of undernutrition in Ethiopia [16, 21–26].

The World Health Organization (WHO) developed two broad strategies to combat undernutrition globally: nutrition-sensitive and nutrition-specific. Nutrition-specific methods address undernutrition’s underlying causes, including insufficient dietary intake of hematological nutrients like iron or vitamin A, supplementation, and the availability of fortified foods. Dietary diversity is a technique meant to promote the availability, accessibility, and utilization of regionally available and acceptable foods that have high micronutrient content and bioavailability year-round [27]. The Ethiopian government has developed various efforts to combat undernutrition among pregnant women, such as promoting nutritional diversification and iron and folic acid supplements, malaria prevention and treatment, the use of bed nets, and deworming pills [28]. Furthermore, the government has made significant efforts to reduce maternal undernutrition by establishing and implementing a nutrition and food strategy, program, and policy. Furthermore, the government created the "Seqota Declaration" to ensure year-round 100% access to sufficient food throughout the country by 2030 [29]. Moreover, health extension programs, notably those concentrating on community-wide nutrition education, are regarded as the most important measures that can help to ensure appropriate nutrition throughout the first 1000 days [28].

Despite all governmental commitments, initiatives, programs, policies, and efforts, the prevalence of undernutrition among pregnant women was significant across the country, especially in rural areas [11, 16, 21–26]. Besides, there were significant regional and urban/rural variations in the prevalence of undernutrition at the national level [11], this implies that more research is needed regarding the prevalence of undernutrition in local settings. Previous research on the prevalence of undernutrition among pregnant women in Ethiopia, however, concentrated exclusively on individual-level factors, with little attention paid to household, community, and context-specific determinants. Furthermore, no evidence regarding the prevalence and determinants of undernutrition has been identified in the current study setting among the stated population group. Moreover, lack of comprehensive evidence in Ethiopia to design the most efficient and effective strategies to reduce the burden of undernutrition among pregnant women by integrating individual, household, and community-level determinants. Findings of this study are very useful to inform implementers, health managers, and decision/policy makers to design comprehensive intervention strategies. Therefore, this study aimed to describe the prevalence and identify determinants of undernutrition among pregnant women in the Hawela Lida district of the Sidama region, Ethiopia.

## Methods

### Study area

This study was conducted in the Hawela Lida district of the Sidama region of Ethiopia. Hawela Lida district is one of the 36 districts in the Sidama region and is located 289 km away from Addis Ababa, the capital city of the country. It has 11 rural and 2 urban *kebeles* (the lowest administrative unit in Ethiopia). Based on the Sidama Regional State Health Bureau 2023 Report, the district has an overall population of 129,949 with an estimated total of 23,928 households. Of these, 24.30% were women of reproductive age (WRA) [30, 31]. Agriculture is the major source of income-producing activity in *kebeles*. The main crops grown in the district are *enset*, maize, coffee, khat, barely, haricot beans, corns, sweet potatoes, and local varieties of cabbage. The district administration has a total of 425 health professionals of different disciplines, 20 health posts, and 4 health centers owned by the government; additionally, there are 5 private medium and two non-governmental (NGO) clinics, and 6 private drug stores. The health post is managed by health extension workers (HEWs) and provides services such as health education, nutritional screening and education, and treatment for children, pregnant women, etc. [32]. The potential health service coverage of the district by health facilities was 90% [33]. The common health problems in the districts are typhoid fever, typhus, undernutrition, diarrhea, malaria, respiratory tract infection, parasitic infection, sepsis, tuberculosis, human immune deficiency, and traffic road accidents, as per the report of the 2023 Sidama Regional State Health Bureau [30, 31].

### Study design and period

A community-based cross-sectional survey was conducted from June 1–25, 2024, among a sample of 515 randomly selected pregnant women.

### Study population

The source population was all pregnant women who were less than 3 months of gestational age and resided in the district.

### Study population

The study population was all randomly selected pregnant women who were less than 3 months of gestational age and had resided in the district for at least 6 months.

### Inclusion and exclusion criteria

All randomly selected pregnant women who have less than 3 months of gestational age and have resided in the district for at least 6 months were included in this study. The women development team leaders and health extension workers conducted house-to-house censuses of all eligible houses to see if pregnant women lived there. A two-stage screening approach was used to identify pregnant women. Women were first interviewed regarding pregnancy symptoms and signs. Women who mentioned symptoms and signs of pregnancy underwent additional screening, which involved a human chorionic gonadotropin (HCG) urine test. We conducted the HCG test for all women who had missed their menstrual cycle for 6 weeks or more. Women were enrolled in this study if the test findings were positive for pregnancy. This screening and enrolling of pregnant women less than 3 months of pregnancy was primary done for the purpose of our larger interventional study, which is ongoing, and the study protocol was registered at ClinicalTrials.gov with registration number NCT06536153. Pregnant women who had a severe illness during the data collection period were excluded from this study due to their inability to communicate. Further, pregnant women who have temporarily resided in the district were excluded.

### Sample size calculation

The sample size was computed using OpenEpi by considering the anticipated prevalence of undernutrition (19.3%), according to the report of a previous study [34], a margin of error of 5%, a 95% confidence level, and a design effect of 2. Based on this information, the minimum required sample size was 240. The minimum required number of clusters was computed by multiplying the effective sample size by the ICC (intraclass correlation coefficient). However, previous studies didn’t report the ICC value. We took the typical value of the ICC to be 0.01 from a range of values (0.01 to 0.05) based on the suggestion of Donner. Accordingly, the minimum needed number of clusters was 240*0.05 = 2.4. However, we have increased the number of clusters to 10 to increase the power of the study. In this study, *kebeles*, which are subsets of districts, were considered clusters. Average cluster size is calculated by dividing the minimum required sample size by the number of clusters. In this case, it was 240/10 = 24.

The design effect was calculated using the formula DEFF = 1+ (n-1)*ICC, where ’n’ is the average cluster size and ICC. In this case, it was 1.23 by inserting in the formula DEFF = 1 + (24–1)*0.01. However, we increased DEEF to 2 to increase the sample size and power of this study. The sample size was adjusted for the non-response rate by adding 10% of the anticipated non-response rate. Based on the above information, the final estimated sample size was 528.

### Sampling technique

**Fig 1:**
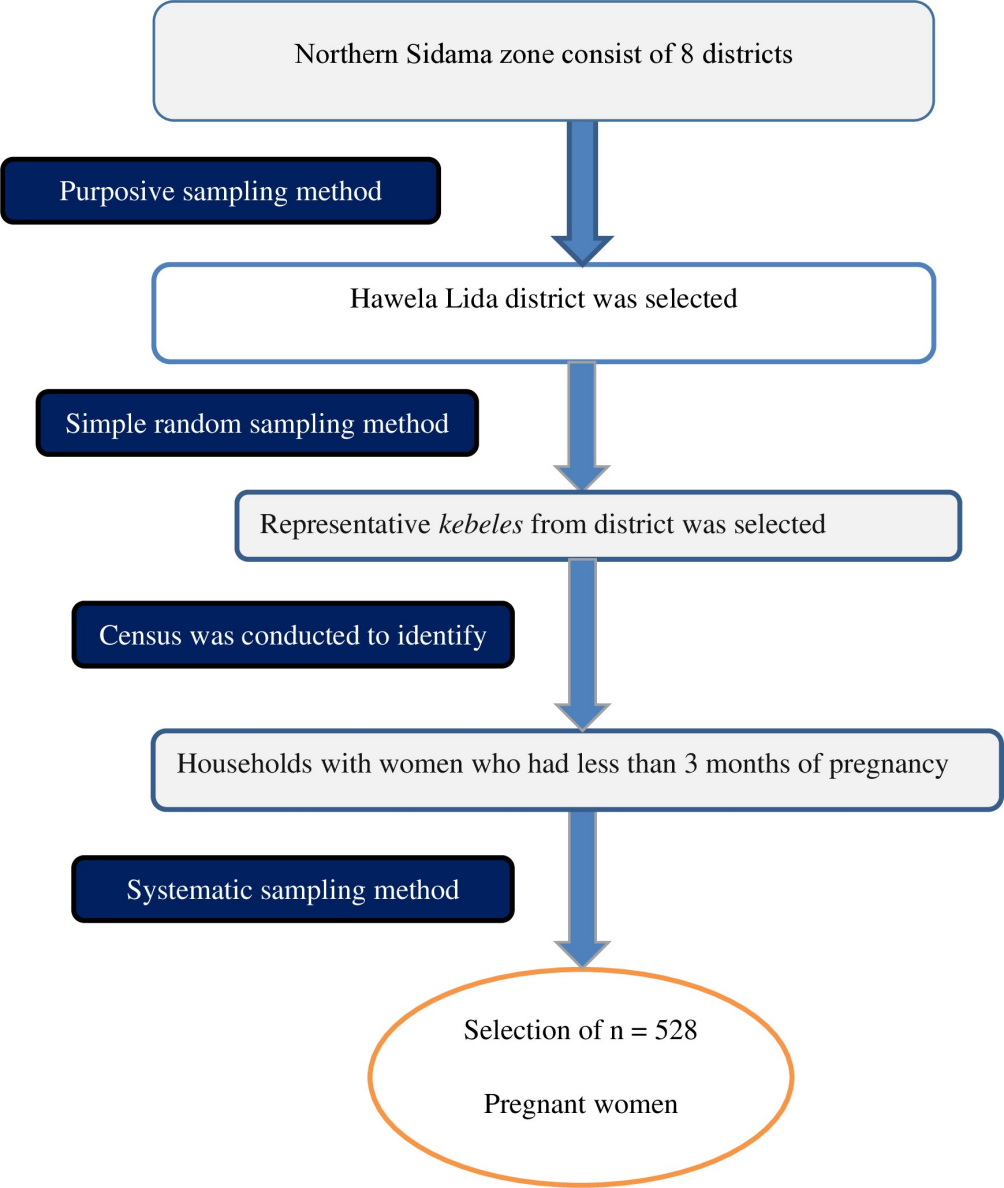
Schematic presentation of the sampling procedure of the study in northern zone of the Sidama region, Ethiopia, 2024.

A multi-stage sampling method was utilized to select the representative pregnant women until the determined sample size was reached. The first stage was the selection of Hawela Lida district from the Sidama region using a purposive sampling method. We selected the district purposefully to facilitate supervision and coordination of our interventional study because, immediately following this baseline survey, our project was designed and implemented. This district is near Hawassa City, which is the capital city of the Sidama region, and provides good geographic access. Second stage was selection of representative *kebeles* from the Hawela Lida district utilizing a lottery sampling technique. Based on the section procedure, 10 *kebeles*, namely Hawela 01, Murancho, Hayisa Wita, Hayisa Baraha, Chafe 01, Galuko Hireye, Gara Galo, Dulacha Tewerako, Hawela Lida rural, and Ramada were randomly selected from the district. The third stage was a selection of households with pregnant women. The household with pregnant women was identified by conducting a house-to-house census, and a sampling frame was prepared. Finally, study respondents were drawn using a systematic sampling technique. Pregnant women which were not available after three consecutive visits were considered as non-respondent for this study. One mother was included by using a lottery sampling procedure when two or more pregnant mothers occur in the chosen households (Fig 1).

### Study variables

The outcome variable was the prevalence of undernutrition. In this study, the mid-upper arm circumference (MUAC) measurements of pregnant mothers who fell below a cutoff point of 23 cm were classified as undernourished, while those who measured 23 cm or more were classified as normal [35, 36]. A flexible, non-stretchable tape was used to measure the mothers’ MUAC by placing them in the Frankfurt plane and observing them sideways to measure the left side arm to the nearest 0.1 cm. The mothers’ arms were hanging lightly at the side, with their palms facing inward.

The independent variables were classified into individual, household and community-level covariates. The individual-level covariates were socio-economic and demographic covariates like women age, occupation of the women and their husbands, education of the women and their husbands; reproductive characteristics such as women age at first marriage and childbirth, gravidity and parity status, history of stillbirth and neonatal death, and pregnancy planning status; knowledge and attitude toward nutrition; and dietary diversity. The household wealth index, family size, mass media use, and household food security were household-level determinants. The community-level covariates are place of residence, distance from the nearest health facilities, community-level road access, community-level literacy rate, community-level women’s autonomy, and community-level poverty.

### Measurement of variables

Dietary diversity scores were calculated using Food and Agriculture Organization (FAO) guidelines [37]. Minimum dietary diversity (MDD-W) for women of reproductive age was aggregated into 9 food groups by looking at the foods and beverages consumed in the previous 24 hours. After computation, the score was divided into two groups, namely adequate and inadequate. Nine food groups were created from all of the pregnant women’s reported foods and drinks consumed the day before the survey: cereals and starchy staples; oils and fats; dark green leafy vegetables and vitamin A-rich fruits and vegetables; legumes; nuts and seeds; other fruits and vegetables; meat and fish; organ meat; milk and products; and eggs. Pregnant women who have eaten the food in each subgroup (at least once) received a score of 1, and otherwise, 0 was assigned.

The household food insecurity questionnaires are based on food and nutritional technical assistance (FANTA) version 3 and have been modified for the local context; they contain 27 questions [38]. The first 9 questions were answered "yes" or "no," and the results were divided into four groups: food secured, mildly, moderately, and severely food insecure. The details of variable measurement are provided in S1 File.

### Data collection tools and techniques

The study tool was a structured and pretested interviewer-administrated questionnaire developed from similar previous studies [39–41]. The questionnaire was initially prepared in English (see S2 File). This tool was translated to the Sidaamu Afoo language (the local language spoken by the local residents) and reconverted back to English to assure similarity between two versions. The translation was conducted by a language expert in both English and Sidaamu Afoo. The translated study tool was reviewed by the principle investigator (PI) and another person who is also an expert in both languages. At that moment, the inconsistency was corrected between the two languages version as per the identified problems. The data collection procedure was managed by 20 data collectors and 4 supervisors. Data were collected from pregnant women at their home using a face-to-face interviewer-administered questionnaire. The PI monitored and controlled the overall process of data collection and made appropriate corrections for any issues.

### Data quality assurance

The training regarding the study tool was provided for the data collectors, field assistant and field supervisors by the PI for 2 days. During the training, attention was given to the importance of the research, the data collection procedure, objectives, sampling procedures, blood sample collection procedures, and ethical considerations. The data collection was conducted using a properly designed, standardized, pretested, structured, face-to-face interviewer-administered questionnaire. After the pre-test, essential adjustments were carried out prior the main data collection process on the tool. The data collection process was carefully supervised. A completeness, consistency and accuracy of data were checked on daily basis during data collection. The data were cleaned, coded, and exported to Stata 17 for further processing and analysis. To minimize the risk of reporting bias, data collectors, field assistants and supervisors were blinded for the exposure and outcome variables. In addition, to minimize the risk of bias, maximum efforts were made by careful selection of subjects that represent the source population, maximizing response, and providing training for the data collectors, field assistants and supervisors.

### Ethics statement

The ethical approval of this study was obtained from the institutional review board (IRB) at the College of Medicine and Health Sciences of Hawassa University with reference number of IRB/027/16. A support letter was obtained from Hawassa University School of Public Health, Sidama region, Hawela Lida district, and *kebeles* leaders. Written consent was secured from pregnant women before data collection and after provision of information about this study. All data collection methods were carried out with confidentiality. Specific personal identifiers were not collected, and only researchers had access to data that could recognize individual respondents during or after data collection. Besides, pregnant women with severe undernutrition detected during the survey were referred to a nearby health facility for further investigation and treatment.

### Data analysis techniques

Before the main analysis, quantitative variables were handled by recoding, calculations, and categorizations. Descriptive analyses were done to get descriptive measures for the important variables of interest, like frequency, percentage, mean, and standard deviation (SD). The Principal Component Analysis (PCA) was carried out for the computation of the wealth index for this study [42] and details of variable preparation and analysis procedures were provided in Table 2 of the S1 File.

We calculated intra-class correlation coefficient (ICC) using a multi-level logistic regression intercept only model [43, 44]. The calculated ICC value was greater than 5% which is one indicator to consider the multi-level analysis regression analysis for this study. Both bi-variable and multivariable analyses using multi-level mixed effects modified Poisson regression models with robust standard error were carried out to examine the effects of clusters and known confounders. Those variables with p-values < 0.25 on the bi-variable analysis and practical significance backed from relevant literature were built-in in a multivariable regression model to find out determinants independently associated with undernutrition, adjusting for other variables in the model [45].

We have developed multi-level models to account for the effects of clustering and the hierarchical nature of our data. Thus, four models were assessed for this study. Model 1 was an empty model or intercept-only model; Model 2 contained only individual-level determinants; model 3 contained only community-level determinants; and the final model (Model 4) contained both individual and community-level determinants. The ICC value and the median prevalence ratio (MPR) were used to assess the random model information [46]. The MPR is a predictor of the unexplained *kebeles*-level heterogeneity, while the ICC value was utilized to characterize the percentage of variability in the prevalence of undernutrition that is attributable to the clustering variable *(kebele)*. The MPR was computed using the formula, MPR = ∼ ${\text{e}}^{0.95*\sqrt{\left(\text{estimated}\;\text{variance}\;\text{of}\;\text{clusters}\right)}}$ and is defined as the mean of the prevalence ratio between the areas at the highest and lowest risk of undernutrition prevalence when two areas are randomly selected [47, 48].

Effect modification and multicollinearity was examined for this study. The variance inflation factor (VIF) < 5 was used to declare that the effect of multicollinearity was minimal enough to affect the findings of this study [49]. The details of the effect modification results were provided in the S1 File.

The strength and presence of a statistically significant relationship between undernutrition and the independent variables were assessed using APRs with a 95% CI and 5% level of significance. A statistically significant relationship between the undernutrition and the independent variables was confirmed when the 95% CI of the APRs did not contain 1 or a P-value less than 0.05.

## Results

### Study participants’ socio-demographic characteristics

**Fig 2:**
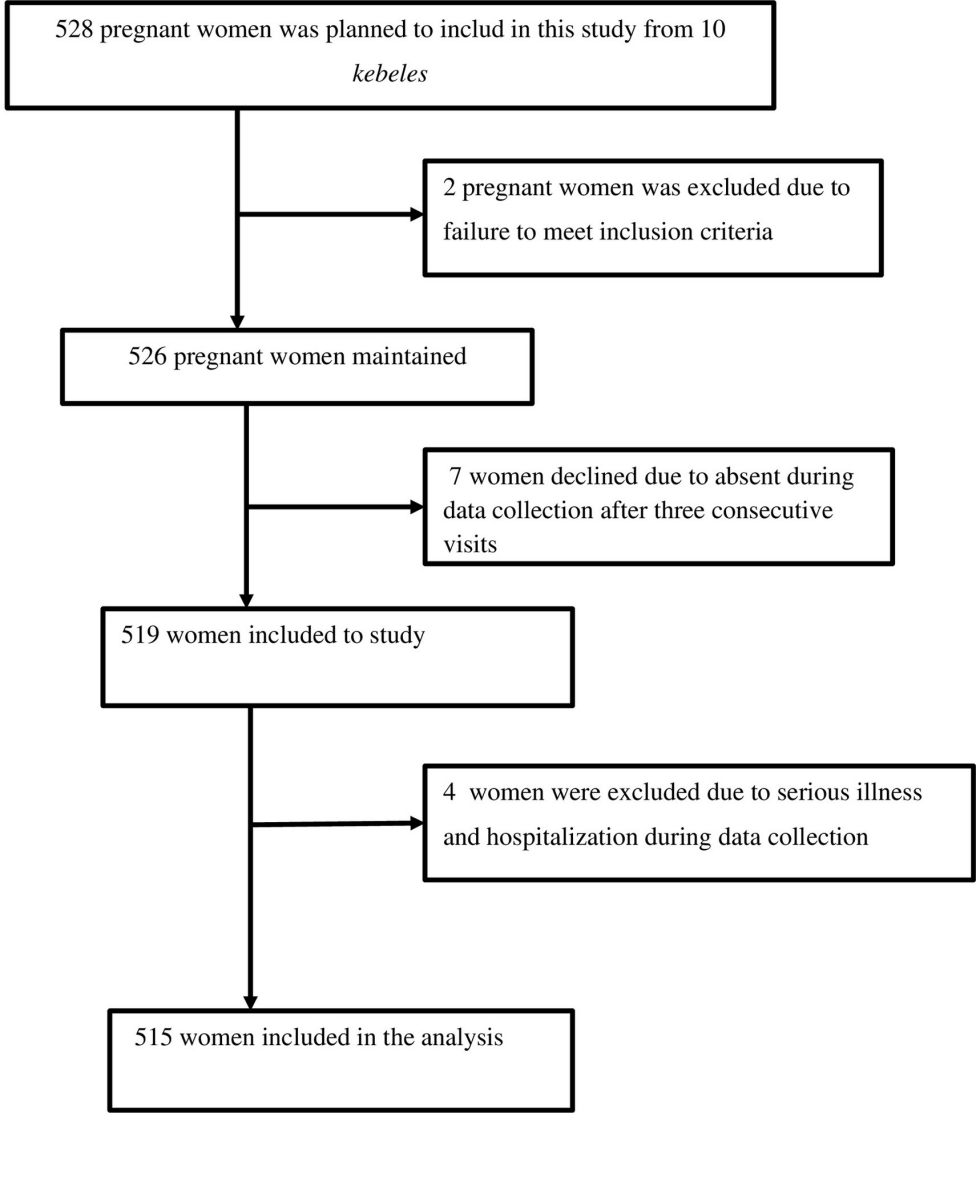
Participants flow diagram for cross-sectional study.

We successfully interviewed 515 pregnant women from 528, for a response rate of 97.53% for this study (Fig 2).

The mean age (+SD) of study participants was 25.89 (+4.53) years. Almost all 486 (94.4%) study participants were of Sidama ethnicity. The majority, 438 (85.2%) of study participants, were Protestant Christian followers. Nearly all, 511 (99.2%) and 468 (90.9%) of pregnant women were married and housewives, respectively (Table 1).

**Table 1:** Socio-demographic characteristics of pregnant women in the Hawela district of Sidama region, Ethiopia, 2024.

Variables	Categories	Frequency (%)
**Ethnicity**	Sidama	486 (94.4)
	Amhara	18 (3.4)
	Wolayita	6 (1.2)
	Gurage	5 (1.0)
**Religions**	Protestant	438 (85.2)
	Orthodox	34 (6.6)
	Catholic	25 (4.9)
	Muslim	14 (2.5)
	Others	4 (0.8)
**Education status**	Illiterate	65 (12.6)
	Can read and write only	105 (20.4)
	Primary education	271 (52.6)
	Secondary education	53 (10.3)
	College diploma	17 (3.3)
	University degree and above	4 (0.8)
**Occupation status**	Housewife	468 (90.9)
	Merchant	30 (5.8)
	Government employee	17 (3.3)
**Education status of husband**	Illiterate	18 (3.5)
	Can read and write only	58 (13.3)
	Primary education	282 (55.2)
	Secondary education	105 (20.5)
	College diploma	23 (4.5)
	University degree and above	15 (2.9)
**Occupation status of husband**	Governmental employee	16 (3.1)
	Merchant	151 (29.5)
	Farmer	311 (60.9)
	Daily laborer	25 (4.9)
	Private organization employee	3 (0.6)
	Others	5 (1.0)
**Marital status**	Married	511 (99.2)
	Divorced	4 (0.8)
**Family size**	Small	376 (73.0)
	Large	139 (27.0)
**Mass media utilization**	Yes	205 (39.8)
	No	310 (60.2)
**Wealth status**	Lowest	103 (20.0)
	Second lowest	106 (20.6)
	Middle	100 (19.4)
	Fourth	99 (19.2)
	Highest	107 (20.8)

**Note:** There were 4 divorced pregnant mothers in this manuscript data. Nevertheless, we collected data from all mothers regarding their husband’s education and occupation status by the period they were in marital union.

### Reproductive health features of study participants

The mean age at first marriage (+ SD) of study participants was 21.01 + 3.10 years. 81 (15.7%) women have a previous history of abortion, and 93 (18.1%) had an infection during their current pregnancy. Similarly, 49 (9.5%) and 28 (5.4%) women have histories of previous stillbirths and neonatal deaths, respectively (Table 2).

**Table 2:** Reproductive characteristics of pregnant women in the Hawela district of Sidama region, Ethiopia, 2024.

Variables	Frequency (%)
	N (%)
**Women’s age during first marriage**	21.01 + 3.10
**Women’s age during first pregnancy**	22.37 + 3.17
**Previous history of abortions**	
No	434 (84.3)
Yes	81 (15.7)
**Infection during the current pregnancy**	
No	422 (81.9)
Yes	93 (18.1)
**Previous history of stillbirth**	
No	466 (90.5)
Yes	49 (9.5)
**Previous history of neonatal death**	
No	487 (94.6)
Yes	28 (5.4)
**Last pregnancy planned**	
No	83 (16.1)
Yes	432 (83.9)

### Prevalence of under-nutrition

**Fig 3:**
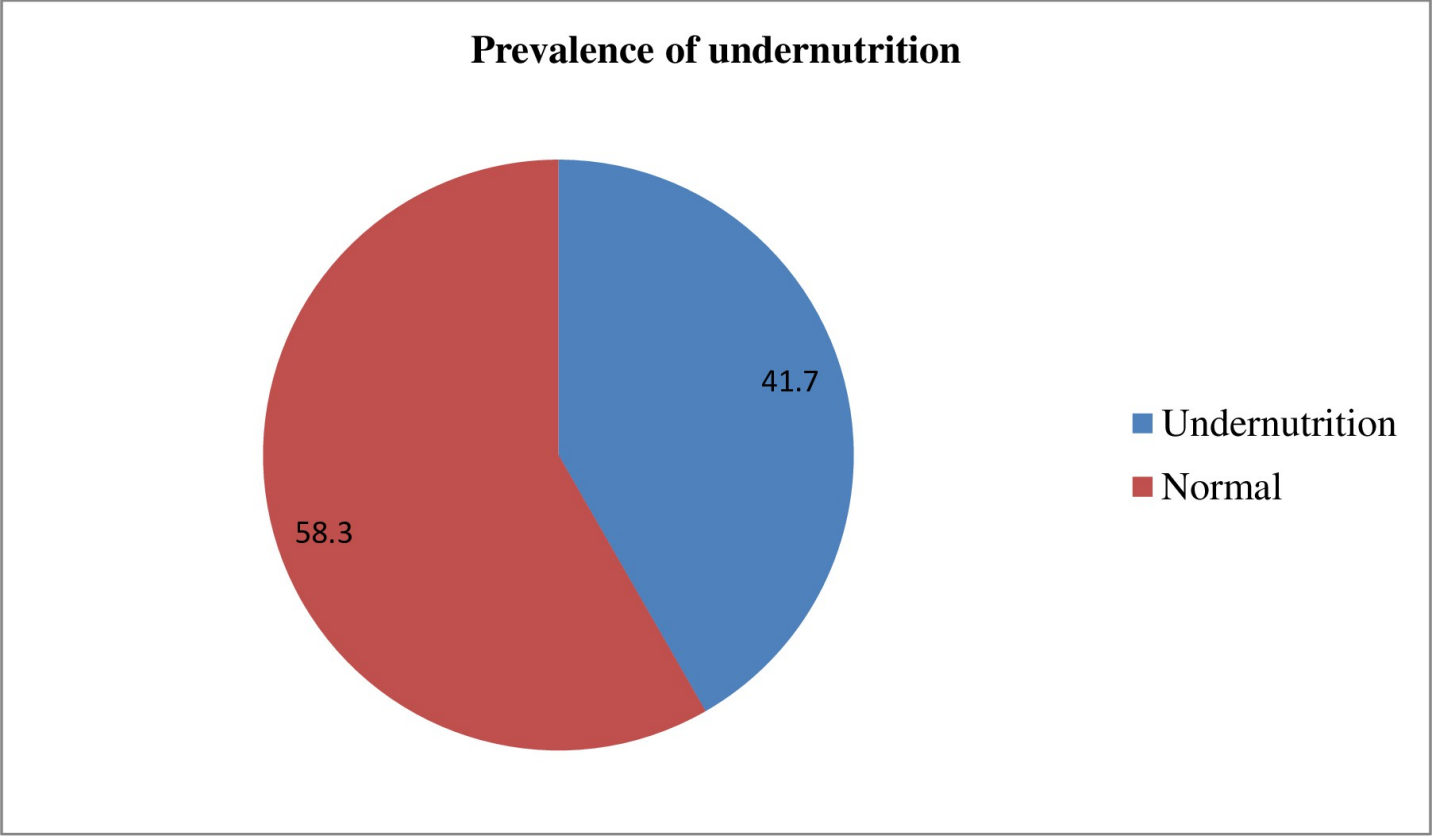
Prevalence of under-nutrition among pregnant women in the Hawela Lida district of Sidama region, Ethiopia, 2024.

The overall prevalence of under-nutrition among pregnant women was 41.7% (95% CI: 37.3–45.6) (Fig 3).

### Determinants of under-nutrition

Pregnant women who had an unplanned pregnancy had a 20% higher likelihood of under-nutrition than their counterparts (APR = 0.80; 95% CI: 0.66–0.98). Pregnant women’s inadequate dietary diversity increased the likelihood of under-nutrition prevalence (APR = 1.79; 95% CI: 1.43–2.25) by 79% compared to women who have adequate dietary diversity. Pregnant women in the food-insecure household had 64% more under-nutrition prevalence as compared to the food-secure household (APR = 1.64; 95% CI: 1.26–2.13). Women who have poor knowledge of nutrition had a higher prevalence of under-nutrition than their counterparts (APR = 1.68; 95% CI: 1.32–2.12). While the low community-level literacy rate increased the likelihood of under-nutrition prevalence (APR = 4.62; 95% CI: 1.13–18.79) as compared to the high community-level literacy rate, the likelihood of under-nutrition prevalence was 91% higher for pregnant women who lived in low wealth status communities (APR = 1.91; 95% CI: 1.10–3.13) as compared to women who lived in high wealth status communities (Table 3).

**Table 3:** Determinants of under-nutrition among pregnant women in the Hawela Lida district of Sidama region, Ethiopia, 2024.

Variables	Nutritional status		CPR (99% CI)	APR (99% CI)
	Under-nutrition	Normal		
**Individual level determinants**				
**Women’s education**				
Have formal education	139 (40.3)	206 (59.7)	Ref	Ref
No formal education	76 (44.7)	94 (55.3)	0.96 (0.87, 1.06)	0.92 (0.83, 1.23)
**Family size**				
Small	156 (38.4)	250 (61.4)	Ref	Ref
Large	59 (54.1)	50 (45.9)	1.10 (0.91, 1.34)	1.11 (0.82, 1.51)
**Mass media utilization**				
Yes	74 (36.1)	131 (63.9)	Ref	Ref
No	141 (45.5)	169 (54.5)	1.05 (0.97, 1.15)	1.11 (0.99, 1.23)
**Wealth quintile**				
Lowest	56 (54.4)	47 (45.6)	Ref	Ref
Second	39 (36.8)	67 (63.2)	1.13 (0.87, 1.48)	1.17 (0.91, 1.50)
Middle	36 (36.0)	64 (64.0)	1.06 (0.83, 1.35)	0.83 (0.59, 1.15)
Fourth	44 (44.4)	55 (56.6)	1.24 (1.04, 1.49)	0.82 (0.58, 1.17)
Highest	40 (37.4)	67 (62.6)	1.19 (0.95, 1.48)	0.78 (0.53, 1.13)
**Infection during current pregnancy**				
No	169 (40.0)	253 (60.0)	Ref	
Yes	46 (49.5)	47 (50.5)	1.17 (0.81, 1.69)	1.01 (0.76, 1.33)
**Last pregnancy planned**				
No	42 (50.6)	41 (49.4)	Ref	Ref
Yes	173 (40.0)	259 (60.0)	0.54 (0.36, 0.81)	0.80 (0.66, 0.98)*
**Decision making power of women**				
Autonomous	105 (33.2)	211 (66.8)	Ref	Ref
Non-autonomous	110 (55.3)	89 (44.7)	2.66 (1.21, 5.83)	1.51 (0.50, 4.50)
**Received model family training**				
No	72 (47.7)	79 (52.3)	Ref	Ref
Yes	143 (39.3)	221 (60.7)	0.62 (0.38, 1.01)	0.76 (0.57, 1.03)
**Food security status**				
Secured households	92 (28.7)	228 (71.3)	Ref	Ref
Insecure households	123 (63.1)	72 (36.9)	1.93 (1.22, 3.06)	1.64 (1.26, 2.13)**
**Dietary diversity status**				
Adequate	64 (26.1)	181 (73.9)	Ref	Ref
Inadequate	151 (55.9)	119 (44.1)	1.94 (1.33, 2.82)	1.79 (1.43, 2.25)**
**Women’s knowledge about nutrition**				
Good	80 (28.2)	204 (71.8)	Ref	Ref
Poor	135 (58.4)	96 (41.6)	2.22 (1.50, 3.29)	1.68 (1.32, 2.12)**
**Women’s attitude towards nutrition**				
Positive	125 (38.2)	202 (61.8)	Ref	Ref
Negative	90 (47.9)	98 (52.1)	1.18 (0.73, 1.87)	1.13 (0.90, 1.41)
**Community-level determinants**				
**Place of residence**				
Urban	18 (19.8)	73 (80.2)	Ref	Ref
Rural	197 (46.5)	227 (53.5)	1.21 (0.82, 1.76)	1.49 (0.76, 2.95)
**Community-level wealth status**				
High	85 (27.9)	220 (72.1)	Ref	Ref
Low	130 (61.9)	80 (38.1)	2.27 (1.13, 4.56)	1.91 (1.10, 3.31)*
**Community-level distance**				
Not big problem	126 (38.7)	200 (61.3)	Ref	Ref
Big problem	89 (47.1)	100 (52.9)	1.30 (0.90, 1.89)	0.74 (0.49, 1.11)
**Community-level literacy**				
High	19 (13.4)	123 (86.6)	Ref	Ref
Low	196 (52.5)	177 (47.5)	7.10 (1.64, 30.58)	4.62 (1.13, 18.79)*
**Community-level road access**				
Accessible	122 (37.9)	200 (62.1)	Ref	Ref
Inaccessible	93 (48.2)	100 (51.8)	1.32 (0.96, 1.82)	1.27 (0.48, 3.39)

APR: adjusted prevalence ratio

*: significant association (*p* < 0.05)

**: highly significant association (*p* <0.01); CI: confidence interval; CPR: crude prevalence ratio; Ref: reference group.

### Random effect model and model fitness information on under-nutrition prevalence

Our assessment showed that the multi-level modified Poisson regression model fit more accurately than the standard Poisson regression model (p <0.001). According to the ICC value, being involved in *kebeles* accounted for 30.15% of the variation in the prevalence of under-nutrition among pregnant women. When two people were randomly chosen from different residential areas, the MPR value showed that the residual heterogeneity between the areas was associated with 1.82 times the individual probabilities of a prevalence of under-nutrition. The final model showed that the variation in under-nutrition prevalence across residential areas remained statistically significant even after correcting for all possible contributing factors.

The prevalence of under-nutrition model fitness evaluation test revealed that the empty model (AIC = 750.10, BIC = 758.59, and log-likelihood = -373.05) was the least fit. However, the models’ fitness improved significantly, especially the final model (AIC = 635.48, BIC = 673.68, and log-likelihood = -308.74). Consequently, when compared to the other models, the final model fits the data the best (Table 4).

**Table 4:** Multi-level modified Poisson regression analysis result of random effect model and model selection information.

Measure of variation	Model 0 (95% CI)	Model 1 (95% CI)	Model 2 (95% CI)	Model 3 (95% CI)
Variance of intercept	0.40 (0.27, 0.59)	0.42 (0.24, 0.72)	0.11 (0.03, 0.48)	0.07 (0.03, 1.67)
ICC percentage	30.15 (14.30–52.75)			
MPR	1.82 (1.63–2.07)			1.28 (1.17, 3.42)
Model fitness				
Log-likelihood ratio	-373.05	-325.65	-335.63	-308.74
AIC	750.10	669.30	683.28	635.48
BIC	758.59	707.50	708.74	673.68

MPR: Median prevalence ratio and ICC: Intra-class correlation coefficient; AIC: Akaike information criteria; BIC: Bayesian information criteria; CI: confidence interval.

## Discussion

The prevalence of undernutrition among pregnant women was 41.7%. Unplanned pregnancy, inadequate dietary diversity, food-insecure status, poor knowledge of nutrition, a low community-level literacy rate, and low community wealth status were determinants of undernutrition among pregnant women.

The prevalence of undernutrition among pregnant women was 41.7%. This finding is in agreement with studies conducted in Tigray region of Ethiopia (40.6%) [50], Konso district of Southern Ethiopia (43.1%) [51], and Eastern Ethiopia (43.8%) [52]. However, this finding is higher than studies done in the Silte Zone (22%) [21], Gambella region (28%) [22], and Gondar of the Amhara region (14%) [53]. Similarly, lower prevalence reported studies conducted in Ghana (11%) [54], systematic reviews from Africa (20%) [55], south Sudan (18.9%) [56], Bangladesh (20%) [57], and Kenya (19.3%) [58]. The varying prevalence of undernutrition in different parts of Ethiopia and other nations could be attributed to differences in socio-demographic factors, degree of economic development, study area, level of health service coverage, and sample size. Our study was focused on rural pregnant women and community-based, whereas the other studies were focused on urban areas and institution-based. Evidence suggests that undernutrition is more prevalent among rural residents.

This research indicated that pregnant women who have planned pregnancy decreased the prevalence of undernutrition. This result is consistent with research from low- and middle-income nations that shows unintended pregnancies can have an impact on health care, child nutrition, and maternal nutrition [59, 60]. This is due to the fact that mothers who have planned pregnancies have programs in place to help them feed themselves and get ready to have a variety of food types in their homes. Besides, a pregnant woman who has planned a pregnancy might have better knowledge of nutrition, be more autonomous in her decision-making, and be well educated.

Pregnant women’s inadequate dietary diversity was significantly related to undernutrition prevalence. Similar findings were described from the studies carried out in the Gambella region, the south Omo zone of Ethiopia, and Keny [22, 24, 58]. This could be because women do not eat extra meals during pregnancy, and maternal dietary habits, socio-cultural beliefs, and food taboos can all have an impact on nutrition during pregnancy. The prevention of malnutrition in all its manifestations, both before and during pregnancy, depends on good nutrition practices, vital nutrition services, and nutritious diets. Thus, it is essential for all prenatal care to provide pregnant women with dietary education and counseling, and this should be increased. Furthermore, the researchers argued that this could be stated as follows: Eating a wide range of foods is essential to getting all the nutrients needed to prevent undernutrition caused by nutrient deficiency [61].

This study indicated that household food-insecure status was positively related with undernutrition prevalence. Studies done in the Arbaminch district of southern Ethiopia [25], Gambella [22], Tigray region of northern Ethiopia [62], and Nepal [63] reported similar findings. This could be because a lack of food in the family typically leads to inadequate daily nutritional needs and low dietary intake, which causes undernutrition in pregnant women. Researchers also argued that one of the main underlying causes of undernutrition is household food insecurity, which arises when a household does not always have physical, social, or financial access to enough food that satisfies their nutritional needs for a healthy life. Furthermore, the women employed coping mechanisms to limit their food intake and provide nourishment for their young children and newborns amid a food scarcity. In light of this, enhancing community food security for households is crucial to preventing and reducing acute undernutrition and its detrimental long-term impacts [64].

Pregnant women’s poor knowledge of nutrition increased the prevalence of undernutrition. This finding is consistent with study results conducted in the Silte zone of southern Ethiopia [21] and Dessie town, northeastern Ethiopia [65]. This might be because inadequate nutritional knowledge about nutrition typically leads to inadequate dietary intake, which causes undernutrition. Also, women who have good knowledge of nutrition may be better able to recognize the advantages of eating a healthy and adequate diet during their pregnancy and be ready to follow a healthy and adequate diet practice.

This study documented that a low community-level literacy rate increased the prevalence of undernutrition. Women with a high community-level literacy rate are more independent and financially independent, have more work possibilities, and are aware of the benefits of proper dietary diversity. Another factor could be that literate populations use more community-level mass media, which may boost the conversation regarding maternal health problems in the community. According to the WHO report, mothers who live in high-income communities may be more exposed to mass media, increasing their awareness and knowledge of nutrition and adequate dietary diversification practices [66]. This notion is further supported by findings from a studies conducted in low-income countries [67, 68].

The prevalence of undernutrition increased for pregnant women who lived in low-income communities. The possible justification for this might be that community-level poverty will decrease the affordability of food. Researchers also discussed that when women have the money to buy these foods, they can access a wider variety of foods, and people’s ability to make purchases influences the kinds of foods they eat [69–71]. The current study’s findings of higher risks of undernutrition among disadvantaged women are in line with those of a prior study conducted on young Ethiopian pregnant women [72]. In underprivileged nations like Ethiopia, women often lack employment opportunities, making it difficult for them to meet their families’ daily needs. Therefore, it stands to reason that funding for women’s economic empowerment would improve pregnant women’s nutritional status.

### Limitations of the study

Some strengths of this study include the community-based nature, the adequate number of study subjects from urban and rural *kebeles* recruited, and accounting for individual, household, and community-level determinants using the multi-level analysis, which is expected to provide representative and comprehensive evidence of undernutrition among pregnant women. This evidence is important to develop relevant policy strategies for effective and efficient reduction of undernutrition among pregnant women. Besides, we measured and accounted for the effect of known potential confounders that can explain the association between the exposure and outcome variables of interest.

However, there are a few fundamental limitations to this study that should be taken into account when interpreting the results. First, the cause-and-effect link between exposures and outcomes cannot be adequately established due to the cross-sectional nature of our study design. Second, because the data used in our study came from the self-report of study participants, it may be susceptible to recall bias. Women may be unable to recall the majority of diet consumed the day before the survey, as well as household food insecurity in the previous four weeks, which may understate the magnitude of their dietary diversity score and food security status, affecting the association with undernutrition. Furthermore, because the data were gathered from women’s self-reports, our findings may have been influenced by reporting bias. There is a risk of purposely misreporting personally relevant determinants such as age, occupation, education, household wealth status, family size, women’s nutrition knowledge and attitudes, dietary diversity, and food security (social desirability bias). As a result, the extent of these factors may have been undervalued or overvalued, and hence their association with undernutrition might have been understated or overstated. Moreover, due to social norms, mothers were reluctant to disclose their state of pregnancy and got an HCG test in the early phases of pregnancy. Yet, this had no substantial effect on our research results. Finally, we haven’t collected the height and weight data for this study to calculate the indicator of chronic nutritional deficiencies (BMI) and triangulate findings with MUAC (wasting).

## Conclusions

The prevalence of undernutrition among pregnant women in the Hawela Lida district of southern Ethiopia is still a severe public health problem. This is an indication that much work remains to be done to improve the nutrition status of pregnant women in the study setting. The health system organization at various levels does not widely address pregnant mothers’ nutritional knowledge to improve their nutritional status. Unplanned pregnancy, inadequate dietary diversity, food-insecure status, poor knowledge of nutrition, a low community-level literacy rate, and low community wealth status were significant determinants contributing to the high prevalence of undernutrition among pregnant women. Thus, any programs related to maternal nutritional improvement strategies should address these determinants that continue to contribute to the high prevalence of undernutrition. Likewise, particularly intervention approaches should be considered for pregnant women with poor knowledge of nutrition, pregnant women who are in unplanned pregnancy, and pregnant mothers who have inadequate dietary diversity. Besides, there is an urgent need to design and enhance the promotion of food security strategies, educate communities with low literacy rates, and create small economic reforms for low-income communities to circumvent the high prevalence of undernutrition.

## Supporting information

S1 File English version study questionnaire. (DOCX)

S2 File Detail information of some methods and result section. (DOC)

S3 File Stata data set. (XLSX)

## List of abbreviations

AIC: Akaike information criteria
APR: Adjusted prevalence ratio
BIC: Bayesian information criteria
CI: confidence interval
CPR: Crude prevalence ration
EDHS: Ethiopian Demographic and Health Survey
FANTA: Food and nutrition technical assistance
FAO: Food and agriculture organization
HCPs: HEW: Health extension worker
MPR: Median prevalence ratio
NGO: Non-governmental organization
PI: Principal Investigator
ICC: Intra-class correlation coefficient
IUGR: Intrauterine growth retardation
IRB: Institutional review board
SD: Standard deviation
WHO: World Health Organization
WRA: Women of reproductive age
VIF: Variance inflation factor
