# Supplementary material for: Individual, household, and community-level determinants of undernutrition among pregnant women in the northern zone of the Sidama region, Ethiopia: A multi-level modified Poisson regression analysis
Source: PLoS One. 2024 Dec 17;19(12):e0315681. doi: 10.1371/journal.pone.0315681 (PMC11651547; doi:10.1371/journal.pone.0315681)
Supplement: S1 File — (DOCX) [file pone.0315681.s001.docx]

**S1 File Table 1:** Description of study variables

| **Individual-level variables** | |
| --- | --- |
| Distance from the HF | is considered as close to HF if a woman is reported to travel less than 5 km or walking hours less than 30 minutes by foot to reach the nearest [[1](#_ENREF_1),[2](#_ENREF_2)]. |
| Use of mass media | is generated by combining whether a study participant listens to the radio, watches television, and reads the newspaper and categorized as “yes” if the respondent is exposed to at least 1 of the 3 media and “no” otherwise [[3](#_ENREF_3),[4](#_ENREF_4)]. |
| Family size | is defined as a total number of individuals existing in the household and is categorized as small when it is < 5, and large (> 5) [[5](#_ENREF_5)]. |
| Women’s knowledge regarding nutrition | was measured using the 16 knowledge questions. The correct answer was assigned 1 score, while the incorrect answer was assigned 0 scores. Hence, the total expected knowledge score ranged from 0 to 16. Hence, the increased total sum of the score was considered to indicate increased knowledge of nutrition on the scale. Finally, it is classified into good and poor using the mean value. Pregnant women who scored below the mean were considered to have poor knowledge. |
| Women’s attitudes regarding nutrition | were assessed using the 15 attitude questions. Each attitude question was measured using a 5-point Likert scale from strongly disagree (1) to strongly agree (5). The total expected attitude score ranged from 15 to 75. Then, the value of each item scored on the Likert scale by respondents was summed, and the value was considered as discrete scale data. Hence, the increased total sum of the score was considered to indicate an increased positive attitude on the scale. Finally, it is classified into positive and negative using the mean value. Pregnant women who scored below the mean were considered to have a negative attitude. |
| Formal education | is the education extending from primary to secondary and higher education and requires an organized and careful purpose that concretizes itself in an official curriculum, applied with a defined calendar and timetable [[6](#_ENREF_6)]. |
| Women’s autonomy | a woman is considered autonomous if a woman can decide when and where to use MHS or on the health care spending by herself alone or with her husband together and a non-autonomous otherwise using a woman’s self-report [[7](#_ENREF_7)]. |
| **Community-level variables** | |
| Place of residence | categorized as urban and rural |
| Community-level women's literacy | the aggregate value of community-level women's literacy was generated by the percentage of women population in the cluster that had at least a primary level of literacy derived from the individual participants’ data. Categorized as a **“**high” concentration of literate women in the *kebeles* if the percentage of women who were at least primary level of education >50% and “low” otherwise [[8](#_ENREF_8),[9](#_ENREF_9)]. |
| Community-level poverty | the aggregate value of community-level poverty was generated by the percentage of households in the cluster in the poorest and poorer quintile derived from the individual participants’ data. Categorized as a **"**high” concentration of poverty in the *kebeles* if the percentage of households in the poorest and poorer quintile >50% and “low” otherwise[[8-10](#_ENREF_8)]. |
| Community-level social media use | the aggregate value of community-level social media use was generated by a percentage of study participants who listens to the radio, watches television and reads the newspaper in cluster derived from the individual participants’ data. Categorized as **“**high” concentration of social media use in the *kebeles* if the proportion of a study participant who uses at least one social media >50% and “low” otherwise[[9](#_ENREF_9),[11](#_ENREF_11)]. |
| Distance from nearest HF | was considered as “close” to HF if a woman reported a walking hour of less than 30 minutes by foot to reach the nearest HFs and “far” otherwise [[2](#_ENREF_2)]. The aggregate value of community-level distance was generated by the percentage of a study participant walking hours to the nearest HF in a cluster derived from the individual participants’ data. Categorized as **“**not big problem” in the *kebeles* if>50% of study participants reported as “close” and “a big problem” otherwise [[2](#_ENREF_2),[12](#_ENREF_12),[13](#_ENREF_13)]. |

**The wealth index** was calculated by using principal component analysis (PCA) as a combined indicator of life standard based on 42 questions related to ownership of prudently selected household assets like the owner of the house, materials used for house construction, the number of rooms in a house, size of agricultural land, presence of herd or farm animals and livestock, types of fuel used for cooking, possession of improved sanitation and water facility [[5](#_ENREF_5),[14](#_ENREF_14)]. The multiple response variables were categorized into binary responses (yes/no) and “I don’t know” responses often coded as 999 to zero (Table 3). Similarly, the "I don't know" response and any missing value are often coded as 999 to zero for the continuous variables [[15](#_ENREF_15)]. The predictors that can differentiate between comparatively "poor" and "rich" households were selected using simple frequency analysis. Thus, our PCA didn’t comprise any assets or variables that were possessed by less than 5% or more than 95% of the individuals in the sample [[2](#_ENREF_2),[15](#_ENREF_15)]. Finally, the component factors or wealth index scores were ranked into 5 classes such as lowest, second-lowest, middle, second-highest, and highest [[5](#_ENREF_5),[14](#_ENREF_14)]. The PCA was carried out for the computation of the wealth index [[14](#_ENREF_14),[15](#_ENREF_15)]. All the basic assumptions of PCA were checked before ranking the components' factor scores into wealth quintiles. We removed the variables from PCA that didn’t satisfy the assumptions such as the Kaiser-Meyer-Olkin (KMO) measure of sampling adequacy less than 0.5, commonalities less than 0.5, and variables that contain the complex structure (high loading correlation >0.4 on greater than one component) [[14](#_ENREF_14),[16](#_ENREF_16)].

**S1 File Table 2:** Some of variables and given values to facilitate the computation of wealth index

| S.no | Variables | Given values |
| --- | --- | --- |
| 1 | Main source of drinking water | Improved: Piped water, tube well or borehole, protected well, protected spring = 1  Unimproved: Unprotected well, Unprotected spring, Lake/pond/stream/canal = 0 |
| 2 | Main source of water used for other purposes such as cooking and hand washing | Improved: Piped water, tube well or borehole, protected well, protected spring = 1  Unimproved: Unprotected well, Unprotected spring, Lake/pond/stream/canal, Surface water (River/dam) = 0 |
| 3 | Where is that water source located? | In own dwelling or yard/plot = 1  Elsewhere = 0 |
| 4 | Type of toilet facilities | Improved: comprise any non-shared toilet of the subsequent kinds: pour/flush toilets to septic tanks, piped sewer systems, and pit latrines; pit latrines with slabs; ventilated improved pit (VIP) latrines; and composting toilets = 1  Unimproved: Pit latrine without slab/open pit, bucket toilet and hanging toilet = 0 |
| 5 | Where is this toilet facility located? | In own dwelling or yard/plot = 1  Elsewhere = 0 |
| 6 | Type of fuel the household mainly use for cooking | Clean fuels include electricity, liquefied petroleum gas (LPG), natural gas, and biogas = 1  Solid fuels include coal, charcoal, wood, straw/shrub/grass, agricultural crops, and animal dung = 0 |
| 7 | Where is the cooking usually done? | In the house and outdors = 0  In a separate building = 1 |
| 8 | Who is the owner of the house? | Me = 1  Rental, family, and relative = 0 |
| 9 | Main material of the roof of the house | Natural roofing (no roof, mud, and sod) = 0  Rudimentary and finished roofing = 1 |
| 10 | Main material of the floor of the house | Natural floor (Earth/sand, dung) = 0  Rudimentary and finished floor = 1 |
| 11 | Main material of the wall of the house | Natural walls (no walls, cane/palm/trunks/bamboo/ree, dirt) = 0  Rudimentary and finished wall = 1 |
| 12 | All other categorical variables were considered as yes and no form | Yes = 1 and no =0 |
| 13 | All continuous variables were treated as continuous |  |
| 14 | “I don’t know” response often coded as 999 for categorical variables | 999 = 0 |
| 15 | “I don’t know” response and any missing value often coded as 999 to zero | 999 and missing value = 0 |

**Effect modification result**

We entered the interaction terms in the final model for women's education and women's knowledge of nutrition, dietary diversity and food security status, community-level wealth status and community-level literacy rate, community-level distance to nearest health facilities, and community-level road access to see if women's education modifies the effect of women's knowledge of nutrition and if food security modifies the effect of dietary diversity, etc. None of the interaction terms was statistically significant, implying the absence of a significant effect modification.

**References**

1. Zegeye K GA, Melese T, (2014) The Role of Geographical Access in the Utilization of Institutional Delivery Service in Rural Jimma Horro District, Southwest Ethiopia . Primary Health Care 4: 150. doi:10.4172/2167- 1079.1000150.

2. Ahmed R SM, Abose S, Assefa B, Nuramo A, Alemu A, et al, (2022) Levels and associated factors of the maternal healthcare continuum in Hadiya zone, Southern Ethiopia: A multilevel analysis. PLoS ONE 17(10): e0275752. https://doi.org/10.1371/journal.pone.0275752

3. Singh P, Singh KK, Singh P (2021) Maternal health care service utilization among young married women in India, 1992–2016: trends and determinants. BMC Pregnancy and Childbirth 21: 122.

4. Fatema K (2019) "Impact of Mass Media on the Utilization of Maternal Healthcare Services in South Asia" (2019). Electronic Theses and Dissertations. 2031. https://digitalcommons.memphis.edu/etd/2031

5. Central Statistical Agency (CSA) [Ethiopia] and ICF (2019) Mini Ethiopia Demographic and Health Survey 2019: Key Indicators Report. Addis Ababa, Ethiopia, and Rockville, Maryland, USA. CSA and ICF. 2019.

6. Shudura E, Yoseph A, Tamiso A (2020) Utilization and predictors of maternal health care services among women of reproductive age in Hawassa University health and demographic surveillance system site, South Ethiopia: a Cross-Sectional Study. Advances in Public Health 2020: 1-10.

7. Asefa A, Gebremedhin S (2019) Mismatch between antenatal care attendance and institutional delivery in south Ethiopia: A multilevel analysis. 9: e024783.

8. Negash WD, Fetene SM (2022) Multilevel analysis of quality of antenatal care and associated factors among pregnant women in Ethiopia: a community based cross-sectional study. 12: e063426.

9. Huda TM, Chowdhury M, El Arifeen S, Dibley MJ (2019) Individual and community level factors associated with health facility delivery: A cross sectional multilevel analysis in Bangladesh. PLoS One 14: e0211113.

10. Liyew AM, Teshale AB (2020) Individual and community level factors associated with anemia among lactating mothers in Ethiopia using data from Ethiopian demographic and health survey, 2016; a multilevel analysis. BMC Public Health 20: 775.

11. Tessema ZT, Animut Y (2020) Spatial distribution and determinants of an optimal ANC visit among pregnant women in Ethiopia: further analysis of 2016 Ethiopia demographic health survey. BMC Pregnancy Childbirth 20: 137.

12. Chaka EE (2022) Multilevel analysis of continuation of maternal healthcare services utilization and its associated factors in Ethiopia: A cross-sectional study. PLOS Glob Public Health 2(5): e0000517. https://doi.org/10.1371/journal.pgph.0000517.

13. Zegeye B, Olorunsaiye CZ (2021) Individual/Household and Community-Level Factors Associated with Child Marriage in Mali: Evidence from Demographic and Health Survey. 2021: 5529375.

14. Vyas S, Kumaranayake L (2006) Constructing socio-economic status indices: how to use principal components analysis. Health Policy Plan 21: 459-468.

15. Fry K. FR, Chakraborty N.M, (2014) Measuring Equity with Nationally Representative Wealth Quintiles. Washington, DC: PSI. .

16. Principal component analysis Available online from https://slideplayer.com/slide/4238108/
